# Impact of community mask mandates on SARS-CoV-2 transmission in Ontario after adjustment for differential testing by age and sex

**DOI:** 10.1093/pnasnexus/pgae065

**Published:** 2024-02-12

**Authors:** Amy Peng, Savana Bosco, Alison E Simmons, Ashleigh R Tuite, David N Fisman

**Affiliations:** Dalla Lana School of Public Health, University of Toronto, 155 College Street, Toronto, ON M5T 3M7, Canada; Dalla Lana School of Public Health, University of Toronto, 155 College Street, Toronto, ON M5T 3M7, Canada; Dalla Lana School of Public Health, University of Toronto, 155 College Street, Toronto, ON M5T 3M7, Canada; Dalla Lana School of Public Health, University of Toronto, 155 College Street, Toronto, ON M5T 3M7, Canada; Centre for Immunization Programs, Public Health Agency of Canada, 130 Colonnade Road, Ottawa, ON K1A 0K9, Canada; Dalla Lana School of Public Health, University of Toronto, 155 College Street, Toronto, ON M5T 3M7, Canada

## Abstract

Mask use for prevention of respiratory infectious disease transmission is not new but has proven controversial during the SARS-CoV-2 pandemic. In Ontario, Canada, irregular regional introduction of community mask mandates in 2020 created a quasi-experiment useful for evaluating the impact of such mandates; however, Ontario SARS-CoV-2 case counts were likely biased by testing focused on long-term care facilities and healthcare workers. We developed a regression-based method that allowed us to adjust cases for under-testing by age and gender. We evaluated mask mandate effects using count-based regression models with either unadjusted cases, or testing-adjusted case counts, as dependent variables. Models were used to estimate mask mandate effectiveness, and the fraction of SARS-CoV-2 cases, severe outcomes, and costs, averted by mask mandates. Models using unadjusted cases as dependent variables identified modest protective effects of mask mandates (range 31–42%), with variable statistical significance. Mask mandate effectiveness in models predicting test-adjusted case counts was higher, ranging from 49% (95% CI 44–53%) to 76% (95% CI 57–86%). The prevented fraction associated with mask mandates was 46% (95% CI 41–51%), with 290,000 clinical cases, 3,008 deaths, and loss of 29,038 quality-adjusted life years averted from 2020 June to December, representing $CDN 610 million in economic wealth. Under-testing in younger individuals biases estimates of SARS-CoV-2 infection risk and obscures the impact of public health preventive measures. After adjustment for under-testing, mask mandates emerged as highly effective. Community masking saved substantial numbers of lives, and prevented economic costs, during the SARS-CoV-2 pandemic in Ontario, Canada.

**Significance Statement**

Mask use for prevention of respiratory infectious disease transmission is not new but has proven controversial during the SARS-CoV-2 pandemic. In Ontario, Canada the irregular introduction of community mask mandates by 34 regional health authorities created a quasi-experiment useful for evaluating mask effects, but such effects could be obscured by under-testing of younger individuals, relative to elders. We compared mask mandate effect estimates based on reported case data and based on case data adjusted for under-testing. While mask mandate effects were variably significant with unadjusted case counts, they were large and robust when test-adjusted counts were used. We estimate that community mask mandates saved thousands of lives, and hundreds of millions of dollars in care costs, in Ontario in 2020.

## Introduction

The use of masks and respirators for prevention of respiratory disease transmission is not new (1–3). However, during the SARS-CoV-2 pandemic the use of these tools has proven controversial (4) and even politically polarizing (5). Supporters and opponents of community masking can point to a mixed evidentiary database when it comes to the impact of masking for reducing disease risk (6). Challenges in assessment of mask effectiveness in community settings include potential confounding in observational studies, and unintended crossover and contamination in randomized trials (7), and the difficulty in teasing apart the bidirectional impacts of masking (prevention of inhalation of viral particles as well as reduction in infectious aerosol generation in infective individuals) (8).

Masking directives were typically issued at times of increased community risk, which may again serve to obscure the impact of masking. Lastly, studies that rely on surveillance data, and evaluate masking using quasi-experimental designs, may be limited by the differential and targeted use of PCR testing in different groups in the population. For example, we previously found that testing in the Canadian province of Ontario tended to be heavily targeted towards females aged 80 and over, who constitute the majority population in the province's long-term care homes (9). We found that adjustment for differential testing by age and sex resulted in a different picture of infection risk by age, with infection risk strongly concentrated in younger age groups once differential testing was accounted for.

In the Canadian province of Ontario, there was no province-wide masking directive introduced in the summer of 2020. Rather, individual public health regions introduced mask mandates for indoor public settings between 2020 June and September, with variable timing. This created an ideal quasiexperiment that permitted evaluation of the effects of community-level masking mandates on disease incidence. An econometric analysis was performed on these data by Karaivanov et al., who found that community masking likely had an efficacy of 22% in reducing SARS-CoV-2 transmission in Ontario (10). However, we hypothesized that the true impact of masking might be obscured by differential testing of the Ontario population by age and sex, with the impact of masking in younger age groups obscured by under-testing.

Our primary objectives were to evaluate the impact of community mask mandates in the period from 2020 March to December in the province of Ontario, using both reported SARS-CoV-2 case time series, and using time series adjusted for differential testing. A secondary objective was to estimate the SARS-CoV-2 prevented fraction for community masking in Ontario during this time period.

## Methods

### Data sources

We evaluated mask mandate impact using population-based SARS-CoV-2 infection data from the Ontario Case and Contact Management System, a data system used by Ontario public health units for public health management of notifiable diseases. The period of interest was from 2020 March 20, when community transmission of SARS-CoV-2 had clearly begun in Ontario, to 2020 December 8. Our reason for ending our analysis in early 2020 December was 3-fold: vaccination against SARS-CoV-2 was initiated in mid-December 2020 (11, 12); more centralized public health guidance for the province around SARS-CoV-2 came into force in 2020 December (13); and 2020 December marked the appearance of the first SARS-CoV-2 variants of concern with the N501Y mutation circulating in Ontario (14). Case count data were available as weekly time series, with counts stratified by 10-year age category, reported case gender (dichotomized as female/non-female), and public health unit (9). Data on weekly PCR test count for SARS-CoV-2 by 10-year age category, reported case gender and public health unit were derived from the Ontario Laboratory Information System (9), and population denominators were obtained from Statistics Canada (15).

Testing rates were evaluated graphically and statistically using negative binomial regression. As highest test rates were seen in females aged 80 and over, we used meta-regression-based methods to adjust case counts in other age and gender groups for under-testing, estimating the case rates that would have been expected if these groups were tested at the same rate as females aged 80 and over. This method is described in detail elsewhere (9) but briefly requires that a standardized infection ratio (SIR), and standardized testing ratio (STR), be estimated weekly, by public health unit, for each age and gender group, with incidence and testing rates in females aged 80 and over used in the denominator of these ratios. It is then possible to create age-, gender-, and public health unit-specific meta-regression models using log-transformed of age (*i*), gender (*j*) and public health unit (*k*) groups, *E*(ln(SIR*_ijk_*)) = *α_ijk_* + *β*(ln(STR*_ijk_*)). As ln(STR*_ijk_*) is zero when a given age and gender group in a given public health unit is tested at the same rate as females aged 80 and over, the model intercept *α_ijk_* can be interpreted as the SIR that *would be expected* in the presence of equal testing. This SIR can then be multiplied by observed infection incidence in females aged 80 and over to generate an estimate of test-adjusted incidence. Weekly test-adjusted case estimates for each public health unit were generated in this way, and used as exposures in models as described below.

### Mask mandate effectiveness

We evaluated mask mandate effectiveness through construction of negative binomial regression models with public health unit populations used as offsets using two different dependent variables: reported weekly case counts and test-adjusted weekly case counts. We represented underlying time trends using a polynomial trend term, in order to capture nonlinear time trends (16). We used a cubic polynomial for the flexibility such a polynomial provides for modeling temporal trends; the full polynomial (including linear and quadratic terms) was incorporated into the model. Other covariates included in the model included age group (modeled as an ordinal variable), gender and provincial reopening stages for each health unit. As SARS-CoV-2 cases declined over the summer of 2020, the government employed a regional, staged approach to reopening, with progressively increasing venue occupancy and gathering sizes, depending on disease activity at the local public health unit level (17). Briefly, stage one reopening applied to some selected workplaces, or those able to modify operations to reduce contacts, and also allowed small gatherings. Stage two reopening increased openings of workplaces and outdoor spaces and permitted gatherings larger in size than in stage one. Stage three was to include reopening of all workplaces with ongoing restriction of large gatherings (17).

We then added community masking mandates to these base models. The effects of staged reopening and mask mandates were considered to have taken effect 1 week after reopening, as the approximate generation time of 5 days for SARS-CoV-2 in 2020 (18–20) would mean that mask mandate and reopening effects would not be seen until the subsequent week in this weekly dataset.

In our primary analysis (Model 1), we fit negative binomial regression models with public health units treated as indicator (dummy) variables. We added community mask mandates to models and evaluated change in model fit with and without mask mandates using both the likelihood ratio test and the *P*-value for the mask mandate coefficient. Incidence rate ratios were estimated for mask mandates, with confidence intervals adjusted for clustering by public health unit. Model 1 can be represented as follows:


$$\begin{array}{rl} \log\;(E(\mathrm{incidenc}e_{ijk})) & =\;\alpha_{ijk}+\;\beta_{\mathrm{time}}(\mathrm{time})+\beta_{t}(t)+\;\beta_{t^{2}}(t^{2})+\;\beta_{t^{3}}(t^{3})\\ & \;+\;\beta_{\mathrm{age}}(\mathrm{ag}e_{i})+\;\beta_{\mathrm{gender}}(\mathrm{gende}r_{j})+\;\beta_{\mathrm{PH}U_{k}}(\mathrm{PH}U_{k})\\ & \;+\overset{3}{\underset{l=1}{\sum }}\;\beta_{\mathrm{reopenin}g_{l}}(\mathrm{reopenin}g_{(t-1)l})+\beta_{\mathrm{mask}}^{*}(\mathrm{mas}k_{\left(t-1\right)}), \end{array}$$

where $E(\mathrm{incidenc}e_{ijk})$ is expected incidence in the *i*^th^ age group, *j*^th^ gender, and *k*^th^ week *t*. The model term $\alpha_{ijk}$ is a constant which includes a model intercept as well as a population offset for the *ijk*^th^ population group. The model also includes *l =* 3 reopening stages as described above, and either includes or excludes a mask mandate term, as denoted by the asterisk (*). Both reopening effects and mask mandate effects are based on policy in force in the prior week (*t −* 1).

We evaluated the robustness of our findings in sensitivity analyses, where we repeated our analysis using alternate modeling approaches, including using cross-sectional time series negative binomial (panel data) approach, with public health units treated as fixed effects and confidence intervals estimated via bootstrapping (Model 2). Model 2 can be represented as follows:


$$\begin{array}{rl} \log\;(E(\mathrm{incidenc}e_{k})) & =\alpha_{k}^{\prime }+\beta_{\mathrm{time}}(\mathrm{time})+\beta_{t}(t)+\beta_{t^{2}}(t^{2})+\beta_{t^{3}}(t^{3})\\ & \;+\overset{3}{\underset{l=1}{\sum }}\;\beta_{\mathrm{reopenin}g_{l}}(\mathrm{reopenin}g_{(t-1)l})\\ & \;+\beta_{\mathrm{mask}}^{*}(\mathrm{mas}k_{\left(t-1\right)}). \end{array}$$

The model is similar to Model 1, but as cases are summed across age- and gender groups, age and gender are not included as covariates in the model. Public health units are no longer modeled as indicator variables, but rather are modeled as public health unit fixed effects, which are incorporated into the model intercept $\alpha_{k}^{\prime }$. We were unable to use the likelihood ratio test to compare models with and without mask mandates due to clustering by public health unit, so mask mandate effects were evaluated based on *P*-values for mask mandate coefficients.

In addition, we used a hierarchical generalized linear modeling approach, with time series nested within public health units, which were treated as random effects (Model 3). For Model 3, a Poisson family and log link were used with the gllamm command in Stata 15 (21) as gllamm does not allow the use of the negative binomial family. Model 3 can be represented as follows:


$$\begin{array}{rl} \log\;(E(\mathrm{incidenc}e_{ijk})) & =\;\alpha_{ijk}^{\prime \prime }+\;\beta_{\mathrm{time}}(\mathrm{time})+\beta_{t}(t)+\;\beta_{t^{2}}(t^{2})+\;\beta_{t^{3}}(t^{3})\\ & \;+\;\beta_{\mathrm{age}}(\mathrm{ag}e_{i})+\;\beta_{\mathrm{gender}}(\mathrm{gende}r_{j})\\ & \;+\overset{3}{\underset{l=1}{\sum }}\;\beta_{\mathrm{reopenin}g_{l}}(\mathrm{reopenin}g_{(t-1)l})\\ & \;+\beta_{\mathrm{mask}}^{*}(\mathrm{mas}k_{\left(t-1\right)}), \end{array}$$

where $\alpha_{ijk}^{\prime \prime }$ can be decomposed into $\alpha_{ij}+\;\nu_{k}$, where $\nu_{k}∼N(0,\sigma_{k}^{2})$ represents random deviation of the *k*^th^ public health unit from the overall intercept.

Our regression-based approach implicitly assumes that relative risks associated with model covariates are multiplicative. However, in the context of a communicable disease epidemic, interventions that alter the reproduction number of the disease may result in exponential decay in risk over time, which would mean that the risk reduction associated with an intervention (such as mask mandates) might be dynamic with respect to time. To capture such dynamic effects, we re-ran Model 1 with mask mandate effects treated as a time-varying covariate. This was done by creating a multiplicative interaction term, with the (0,1) covariate for mask mandates multiplied by the model's cubic time trend term. The presence of time-varying mask mandate effect was assessed by evaluating the *P*-value for the interaction term; model fit with and without the interaction terms was assessed using the likelihood ratio test.

Test-adjusted time series were calculated from test rates and case rates and consequently estimates included some uncertainty. We calculated upper- and lower-bound estimates for weekly case counts by multiplying upper- and lower-bound intercepts from metaregression models predicting ln(SIR*_ijk_*) by weekly case counts in the *ijk*^th^ group and summing these. We evaluated the robustness of our model findings in the face of such uncertainty by creating 1,000 synthetic datasets with case counts for each age group, gender, public health unit, and week through random draws from normal distributions based on the mean and standard errors of ln(SIR*_ijk_*). Models were then re-run on each of these 1,000 datasets, and we then calculated the mean and 95% credible intervals for mask mandate effects from these 1,000 model runs. We also performed sensitivity analyses where models were re-fit using upper- and lower-bound estimates for test-adjusted case counts.

### Prevented fraction

We estimated the prevented fraction of test-adjusted infections between first introduction of mask mandates on 2020 June 12 and 2020 December 8 based on Model 1 predictions with mask mandates, and by generating Model 1 predictions with the community mask mandate variable set to zero; we also estimated prevented fractions and 95% confidence intervals using the punaf command in Stata 15 (22). We estimated the health gains associated with cases averted through mask mandates by applying Ontario-derived age-specific case-fatality, hospitalization and ICU admission risk estimates as in (23). For deaths averted, we assigned gains in quality-adjusted life years (QALY) based on the approach of Briggs and Kirwin (24, 25). The economic costs averted due to hospitalizations and ICU admissions averted were assigned based on estimates from the Canadian Institute for Health Information (26); we assigned an economic value of $30,000 to each QALY gained based on the approach of Kirwin et al. (25). Maps were created using QGIS (27), while other statistical analyses were conducted in Stata version 15 (28). Aggregate data files needed for replication of results are available at https://figshare.com/articles/dataset/Data_for_Fisman_et_al_Test-Adjusted_Incidence_of_COVID-19_in_Ontario/14036528. The study was approved by the Research Ethics Board of the University of Toronto (protocol number 00044787).

## Results

**Fig. 1.**
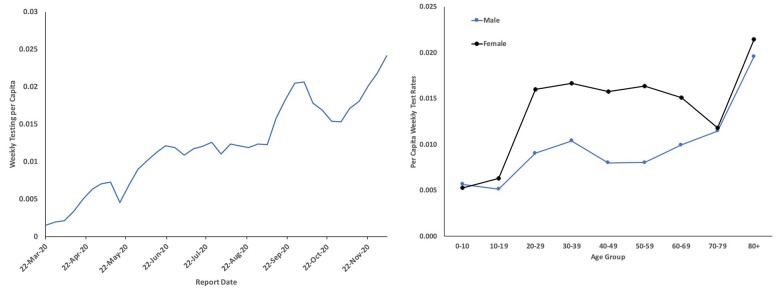
Trends in testing for SARS-CoV-2 in Ontario, Canada, 2020. Weekly per capita tests are presented on the Y-axis in (A) and (B). In A), test report date is presented on the X-axis. Testing increased at an average rate of 4.3% (95% CI 4.2 to 4.4%) from March to December 2020. B) presents weekly per capita tests by age group (X-axis) and sex. Females testing rates are plotted in the upper black curve; the lower blue curve represents testing in males.

There was a significant upward log-linear trend in testing rates over time (relative increase 4.30% per week, 95% CI 4.20 to 4.40%) (Figure 1A). Test rates in females were higher than in males (IRR 1.624, 95% CI 1.590 to 1.660), and test rates in those aged 80 and over were higher than in younger individuals (IRR 1.670, 95% CI 1.613 to 1.730) during the time period under study. In models in which each age and gender group was treated as a unique category, women aged 80 and over were tested at significantly higher rates than all other groups except females aged 20–49, in whom test rates were not significantly different (Figure 1B). As such, we used females aged 80 and over as our referent group for calculation of standardized testing and infection ratios, and for calculation of test-adjusted case counts.

**Fig. 2.**
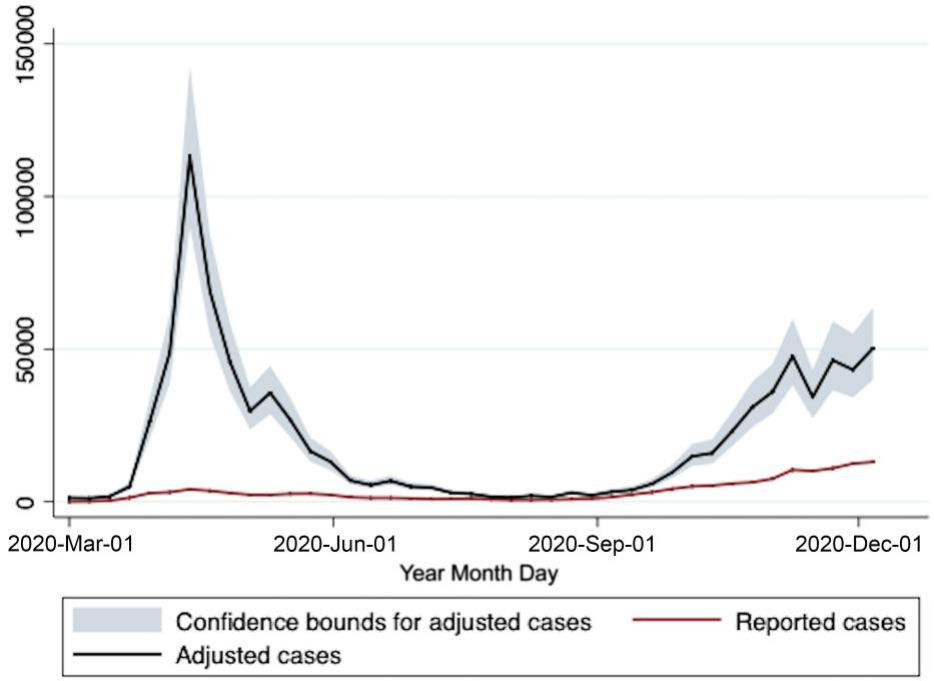
Ontario SARS-CoV-2 epidemic curve in 2020 with and without test adjustment. Weekly case counts are presented on the Y-axis; test report dates are presented on the X-axis. The lower red curve shows reported case counts without adjustment for under-testing. The upper blue curve shows expected case counts if all age and sex groups were tested with the same intensity as females aged 80 and over; the gray shaded area represents upper and lower confidence bounds for test-adjusted cases, estimated as described in the text.

**Fig. 3.**
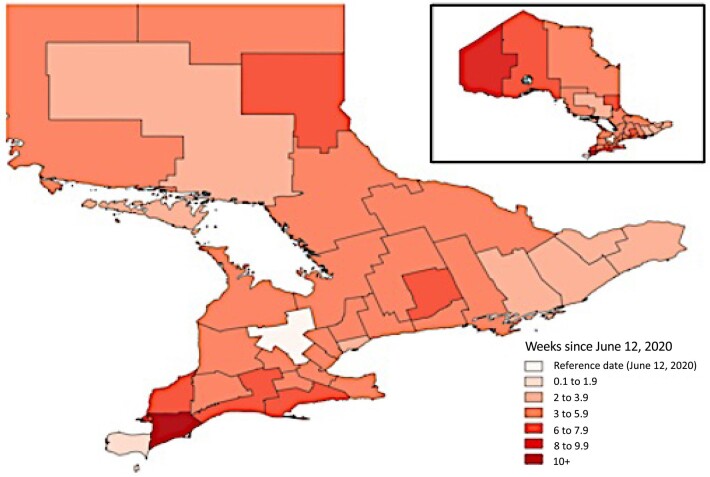
Timing of indoor mask mandates in Ontario, Canada, 2020. Inset shows the entire province, while main body of the map is restricted to southern Ontario. Intensity of color represents the lag since introduction of indoor mask mandates in the Wellington–Dufferin–Guelph public health unit on 2020 June 12. Northwestern health unit (inset, left side of map) and the Chatham–Kent health unit were the last public health units to introduce mask mandates, with Chatham–Kent's mandate introduced on 2020 September 14.

The epidemic curve for SARS-CoV-2 in Ontario, and the epidemic curve created by adjusting for under-testing by age and sex, is presented in Figure 2. While the reported epidemic curve demonstrated highest incidence in autumn 2020, test adjustment revealed a larger spring 2020 wave, with a smaller autumn wave. Introduction of indoor mask mandates by individual public health units began on 2020 June 12 (Wellington–Dufferin–Guelph), with the Chatham–Kent public health unit the last to introduce a mask mandate (2020 September 14) (Figure 3).

We fit negative binomial regression models that included adjustment for time trends, age, female gender, public health unit, and staged reopening for both reported cases and test-adjusted cases. For both datasets, the addition of mask mandates significantly improved model fit (*P* < 0.001 for likelihood ratio test for both models). For both dependent variables, indoor mask mandates were associated with a significant reduction in incidence (for reported cases, IRR 0.687; 95% CI 0.610 to 0.773; for test-adjusted cases IRR 0.512, 95% CI 0.467 to 0.561) (Table 1). The effect of mask mandates was significantly stronger when test-adjusted cases were used as the dependent variable (*P* < 0.001 by Wald test). Mask mandate effectiveness estimates presented in Table 1 are calculated as (1-IRR).

**Table 1.** Estimated effectiveness of mask mandates in reducing SARS-CoV-2 incidence, Ontario, Canada, 2020.

	Dependent variable				
	Reported cases	Test-adjusted cases	Bootstrap sampling (mean and 95% credible interval)	Lower-bound test-adjusted cases	Upper-bound test-adjusted cases
Model 1	31% (23 to 39%)	49% (44 to 53%)	49% (48 to 50%)	51% (44 to 54%)	47% (42 to 51%)
*P*-value, LR test	<0.001	<0.001	—	<0.001	<0.001
*P*-value, mask mandate coefficient	<0.001	<0.001	—	<0.001	<0.001
Model 2	36% (−4 to 61%)	66% (33 to 83%)	66% (66 to 66%)	66% (39 to 81%)	66% (36 to 82%)
*P*-value, mask mandate coefficient	0.073	0.002	—	0.001	<0.001
Model 3	42% (−39 to 76%)	76% (57 to 86%)	75% (74 to 77%)	76% (58 to 87%)	73% (52 to 85%)
*P*-value, LR test	<0.001	<0.001	—	<0.001	<0.001
*P*-value, mask mandate coefficient	0.281	<0.001	—	<0.001	<0.001

Model 1 is a negative binomial regression model with a log link and public health units treated as indicator variables; Model 2 is negative binomial panel data model with public health units treated as fixed effects; and Model 3 is generalized linear model a Poisson regression model with a log link and public health units treated as random effects.

We evaluated the robustness of these findings with alternate modeling approaches. For Models 2 and 3, mask mandates were associated with reduced risk when reported cases were modeled, but these effects were not statistically significant (IRR 0.637, 95% CI 0.390 to 1.042 and IRR 0.576, 95% CI 0.240 to 1.385, respectively). By contrast statistically significant, protective effects were seen with test-adjusted cases (IRR 0.342, 95% CI 0.174 to 0.671 and IRR 0.243, 95% CI 0.140 to 0.425, respectively). Results did not change when we used upper- or lower-bound estimates for test-adjusted cases in sensitivity analyses. Results of all three models, and all outcome variables including upper- and lower-bound cases, are presented as mask mandate effectiveness estimates in Table 1. Recreation of models using 1,000 synthetic datasets generated through random draws of ln(SIR*_ijk_*) resulted in identical mean estimates of effect to those derived via deterministic analyses (Table 1); means and ranges are presented as violin plots in Figure S1.

We explored the possibility that the effects of mask mandates might be modified by urbanicity (Greater Toronto/Hamilton Metropolitan Area vs. elsewhere), older age (60 or older), or gender by adding multiplicative interaction terms to our initial negative binomial model (Model 1). No significant interaction was seen between mask mandate effect and older age (*P* = 0.689), or older age and male gender (*P* = 0.314). However, the effect of mask mandates was significantly stronger within the province's major urban area [Greater Toronto/Hamilton Area (GTHA)] than outside (*P* for interaction < 0.001; IRR within GTHA 0.370, 95% CI 0.332 to 0.412; IRR outside the GTHA 0.533, 95% CI 0.487 to 585).

When we modeled the effect of mask mandates as time varying by incorporating a multiplicative interaction term into the model, we identified statistically significant interaction between mask mandate effect and time (*P* < 0.001); treatment of mandate effect as a time-varying covariate resulted in significantly improved model fit (*P* < 0.001 by likelihood ratio test). The risk reduction associated with mask mandates increased over time, consistent with the expected effects of masks on reduction of the epidemic's reproduction number with exponential decay in risk relative to a scenario where mask mandates were not implemented (Figure S2).

**Fig. 4.**
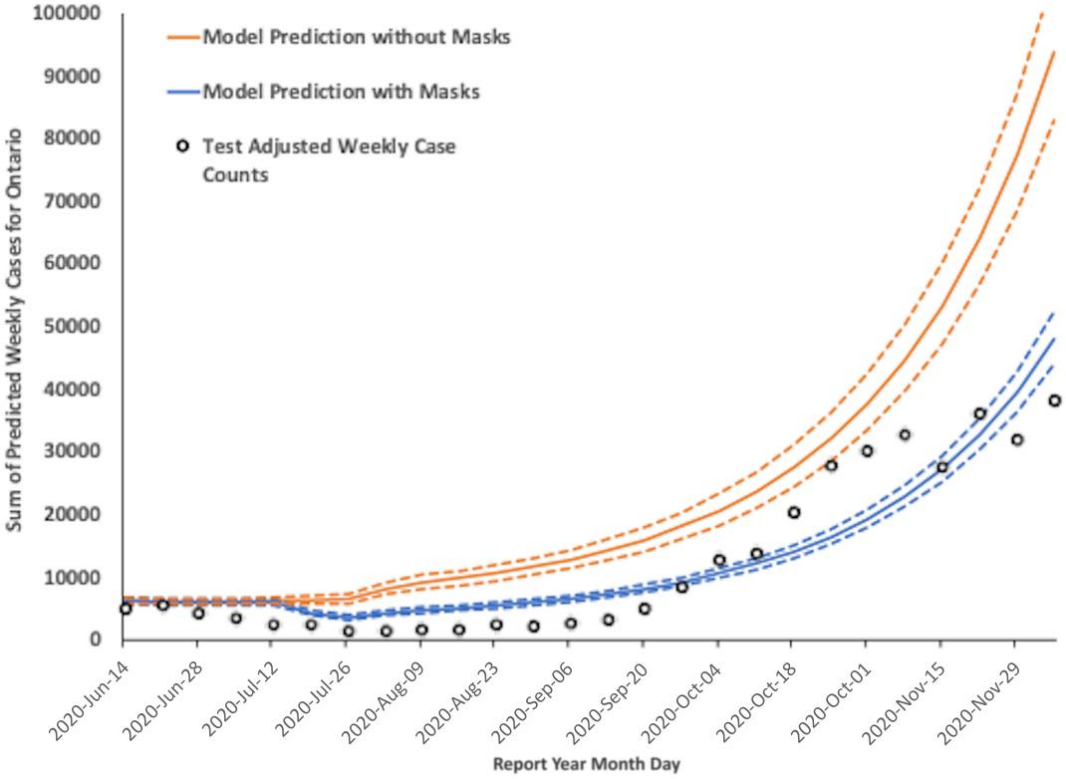
Model-based estimation of mask mandate impact. Test-adjusted weekly case counts (circles) were used to fit a negative binomial regression model that included mask mandate effects (predictions presented in the lower blue curve; dashed curves are 95% confidence intervals). Predictions from a negative binomial model with mask mandate effects set to zero, but all other covariates identical, is shown as an upper orange curve (dashed lines are 95% confidence intervals). The gap between the two modeled curves is the estimated fraction of cases prevented by indoor mask mandates.

We estimated prevented fractions for mask mandates during the period from 2020 June 12 to 2020 December 8 by comparing predictions from Model 1 to predictions from Model 1 with mask mandate effect set to zero (Figure 4). With mask mandates, Model 1 predicted 338,297 cases during this period (as compared to our test-adjusted estimate of 324,311 cases). When mask mandate effect was set to zero, cases rose to 629,137, or 1.860 (95% CI 1.706 to 2.027) times higher than occurred with mask mandates. The prevented fraction due to mask mandates was estimated to be 46.2% (95% CI 41.4 to 50.7%). Based on age-specific hospitalization risk, intensive care admission risk, and case fatality, as well as healthcare costs, we estimated that Ontario's mask mandates prevented 3,008 deaths, 9,546 hospitalizations, 1,879 ICU admissions, and the loss of 29,038 QALY. We estimate that the cost of premature deaths and healthcare usage averted through community mask mandates over this period had a value of approximately $CDN 610 million (Appendix S1).

## Discussion

While the physical properties of masks and respirators reduce both production of infectious aerosols containing SARS-CoV-2 (29), and inhalation of infectious aerosols (30), the application of masks and respirators in indoor settings during the pandemic has been variable (31–34) and controversial (4, 5). Both real-world evidence and randomized trial evidence on the effects of community-level masking have been mixed in strength (6, 10, 35–40). While Ontario's irregular, local introduction of indoor mask mandates established a quasi-experiment ideal for evaluation of mask effects, and earlier work suggests a modest reduction in SARS-CoV-2 incidence was associated with these mask mandates (10), we found that a large and robust effect of mask mandates was seen once data were adjusted for under-testing in younger individuals and in males.

Our findings likely identify an important source of bias towards the null in the available literature on community masking effects. We find that the effects of mask mandates are obscured by disproportionate testing of older individuals who are likely to present with more severe SARS-CoV-2 infection (41), and under-testing of younger individuals (children, teens, and young men) who are less likely to undergo testing, and more likely to experience minimally symptomatic infection (42). Younger individuals are expected to contribute heavily to transmission dynamics, both because of density of social contacts (43), and (among younger men) attitudes towards risk (31). While this would be expected to be an important source of bias towards the null in observational studies (35, 36), it would also be a source of bias in randomized trials with differential identification of symptomatic infection by age such as (39), and indeed may partially explain the finding of greater mask effectiveness in older individuals in that study.

We have evaluated the introduction of mask mandates as a surrogate for the effectiveness of masks against transmission of SARS-CoV-2 in the community. However, while it is expected that the ecological study of mask mandates at a regional level is a reasonable surrogate for community mask effectiveness, this approach will result in bias towards the null due to imperfect compliance with masking in the community. Biases towards the null might also be expected in community-based trials of masking in which masked and unmasked individuals interact. As the bidirectional effects of masking (8) protect wearers and those around them (by acting as “source control”), causing a reduction in the *difference* in risk between wearers and nonwearers. However, our use of a quasiexperimental design with masking treated as a ubiquitous exposure would have reduced the impact of such a bias; in other words, the substantial risk reduction that we report here would be an average reduction accrued by both mask wearers *and* nonwearers in a given jurisdiction.

We identified a significant difference in mask mandate effectiveness in the province's principal urban area (the GTHA) as compared to less urban areas. Possible hypotheses about the mechanism underlying such an effect might include differences in density and contact patterns between urban and less urban areas, or differences in exposure to settings (e.g. subways or large indoor manufacturing facilities) where masks may have been particularly helpful in reducing transmission. Given reported gender differences in compliance with public health measures (31, 32, 34), we evaluated the possibility of the modification of mask mandate effects by gender but found no difference in risk reduction between males and females, which may again reflect the importance of indirect effects of masking that extend beyond the wearer.

Our test adjustment serves as a means to correct for the fact that infection incidence during the SARS-CoV-2 pandemic was strongly determined by rates of clinical testing. The usual gold standard for identification of true infection incidence, as opposed to rates of case identification, is serology. However, in the context of SARS-CoV-2 in Canada, serological data have a number of important limitations, including performance of sero-epidemiological studies in nonrepresentative populations such as blood donors, the imperfect sensitivity and specificity of SARS-CoV-2 serological assays, and challenges in identifying repeated infections, as well as difficulty in differentiating infection from vaccination when anti-S antibody assays are used. Our methodology makes it possible to more accurately evaluate the impact of interventions on infection incidence even when serological data are limited in availability, accuracy, or representativeness.

Like any epidemiological study ours has limitations. Key among these is the question of generalizability of mask mandate effectiveness from Ontario to elsewhere in Canada, or to countries outside Canada. Furthermore, the effects we describe are observed at a time period when the ancestral strain of SARS-CoV-2 was circulating, and prior to the availability of SARS-CoV-2 vaccines and emergence of variants with increased infectivity. We are unlikely to have the opportunity to repeat this work in the current epidemiological context. It is possible that unmeasured confounding could have obscured the true relationship between our exposure of interest (introduction of mask mandates) and outcomes. We note that our work builds on the work of Karaivanov et al. (10), who found that even the relatively modest effect of mask mandates that they identified, using reported case counts, remained robust after adjustment for concomitant nonpharmaceutical disease control measures (such as school closures and international travel restrictions) as well as movement data. Indeed, with respect to potential biases, our approach to estimation of prevented fractions using statistical models has likely resulted in conservative estimates of the impact of mask mandates, as our approach does not capture positive feedback loops that would be driven by accelerating case growth.

In summary, we find that adjustment for under-testing in younger age groups demonstrates that community mask mandates in Ontario, Canada were highly effective, and these effects were robust to different modeling approaches. Community masking mandates generated substantial health and economic benefits for the province. Such mandates should be considered a potent tool for the management of future respiratory virus emergences.

## Supplementary Material

pgae065_Supplementary_Data

## Data Availability

Aggregate data files needed for replication of results are available at https://figshare.com/articles/dataset/Data_and_code_needed_to_recreate_Impact_of_Adjustment_for_Differential_Testing_by_Age_and_Sex_on_Apparent_Epidemiology_of_SARS-CoV-2_Infection_in_Ontario_Canada_/24243181. Questions related to data analytic methods can be directed to Dr. Fisman.
