# Correction to: Impact of community mask mandates on SARS-CoV-2 transmission in Ontario after adjustment for differential testing by age and sex

**DOI:** 10.1093/pnasnexus/pgae098

**Published:** 2024-03-08

This is a correction to: Amy Peng, Savana Bosco, Alison E Simmons, Ashleigh R Tuite, David N Fisman, Impact of community mask mandates on SARS-CoV-2 transmission in Ontario after adjustment for differential testing by age and sex, PNAS Nexus, Volume 3, Issue 2, February 2024, pgae065, https://doi.org/10.1093/pnasnexus/pgae065

The originally published version of this manuscript has been corrected.

In the following sentence in the Results section, the phrase “1,879 hospital admissions” has been corrected to read “1,879 ICU admissions”:

“Based on age-specific hospitalization risk, intensive care admission risk, and case fatality, as well as healthcare costs, we estimated that Ontario's mask mandates prevented 3,008 deaths, 9,546 hospitalizations, 1,879 hospital admissions, and the loss of 29,038 QALY.”

In the following sentence in the Discussion section, the word “prevented” has been corrected to read “generated”:

“Community masking mandates prevented substantial health and economic benefits for the province.”

