# Supplementary material for: Impact of community mask mandates on SARS-CoV-2 transmission in Ontario after adjustment for differential testing by age and sex
Source: PNAS Nexus. 2024 Feb 12;3(2):pgae065. doi: 10.1093/pnasnexus/pgae065 (PMC10923507; doi:10.1093/pnasnexus/pgae065)
Supplement: pgae065_Supplementary_Data [file pgae065_supplementary_data.docx]

**Appendix 1. Estimated SARS-CoV-2-Related Health and Economic Consequences Averted through Community Mask Mandates, June 12-December 8, 2020.**

| **Age Group** | **Test-adjusted Cases** | **Modeled Cases (mask mandate)** | **Modeled Cases (no mask mandate effect)** | **Excess Cases** | **Case Fatality** | **Deaths Averted** | **Hospitalization Risk** | **Hospitalizations Averted** | **ICU Admissions** | **ICU Admissions Averted** |
| --- | --- | --- | --- | --- | --- | --- | --- | --- | --- | --- |
| 0-10 | 43484 | 46231 | 85967 | 39736 | 0.00008 | 3 | 0.00719 | 286 | 0.00056 | 22 |
| 10-19 | 47490 | 46226 | 85952 | 39726 | 0.00004 | 2 | 0.00324 | 129 | 0.00065 | 26 |
| 20-29 | 58665 | 51844 | 96455 | 44611 | 0.00017 | 7 | 0.00637 | 284 | 0.00095 | 43 |
| 30-39 | 45060 | 46601 | 86698 | 40097 | 0.00051 | 21 | 0.01390 | 557 | 0.00247 | 99 |
| 40-49 | 38812 | 44709 | 83150 | 38441 | 0.00138 | 53 | 0.02289 | 880 | 0.00516 | 198 |
| 50-59 | 34389 | 44878 | 83446 | 38568 | 0.00446 | 172 | 0.04138 | 1596 | 0.01080 | 417 |
| 60-69 | 22082 | 31043 | 57707 | 26664 | 0.01684 | 449 | 0.04138 | 1103 | 0.01080 | 288 |
| 70-79 | 17516 | 16640 | 30933 | 14293 | 0.05963 | 852 | 0.18375 | 2626 | 0.04226 | 604 |
| 80+ | 16812 | 10127 | 18830 | 8703 | 0.16644 | 1449 | 0.23955 | 2085 | 0.02099 | 183 |

| **Age Group** | **QALY Loss per Death** | **QALY Gained** | **Non-ICU Hospitalizations** | **Non-ICU Hospitalization Costs Averted** | **ICU Costs Averted** | **Healthcare Costs Averted** | **QALY Valuation†** | **Total Costs Averted** |
| --- | --- | --- | --- | --- | --- | --- | --- | --- |
| 0-10 | 41.37 | 124 | 263 | $5,852,982 | $1,060,868 | $6,913,850 | $3,715,823 | $10,629,673 |
| 10-19 | 37.19 | 56 | 103 | $2,284,035 | $1,235,314 | $3,519,350 | $1,693,147 | $5,212,497 |
| 20-29 | 33.37 | 250 | 242 | $5,369,107 | $2,038,035 | $7,407,143 | $7,494,157 | $14,901,300 |
| 30-39 | 29.40 | 607 | 458 | $10,180,763 | $4,741,782 | $14,922,545 | $18,205,518 | $33,128,062 |
| 40-49 | 24.90 | 1322 | 682 | $15,141,326 | $9,495,830 | $24,637,156 | $39,654,659 | $64,291,815 |
| 50-59 | 20.18 | 3472 | 1179 | $26,199,292 | $19,952,855 | $46,152,147 | $104,174,924 | $150,327,071 |
| 60-69 | 15.36 | 6897 | 815 | $18,113,386 | $13,794,791 | $31,908,177 | $206,903,815 | $238,811,992 |
| 70-79 | 10.35 | 8821 | 2022 | $44,926,962 | $28,920,682 | $73,847,644 | $264,620,983 | $338,468,627 |
| 80+ | 5.17 | 7489 | 1902 | $42,254,736 | $8,745,211 | $50,999,948 | $224,664,993 | $275,664,940 |

**NOTE:** Cases averted estimated as the difference between predictions of model with and without mask mandate effect.

†QALY valued at $30,000 (Canadian dollars).

**Supplementary Figure 1. Mask Mandate Relative Risks in Synthetic Datasets**

The “violin plot” below shows estimated relative risks associated with mask mandates when Models 1-3 were re-run on 1000 synthetic datasets in which weekly test-adjusted case counts by age, gender and public health unit were estimated using random draws from the normal distribution defined by the mean and standard error of ln(SIR_ijk_)). Model 1 is a negative binomial model with public health units treated as indicator variables; Model 2 is a panel data model that treats public health units as fixed effects; and model 3 is a generalized linear multilevel model that treats public health units as random effects. Dark horizontal lines represent mean model results. The width of violin figures denotes the density of model runs for each value of mask mandate-associated relative risk.

**
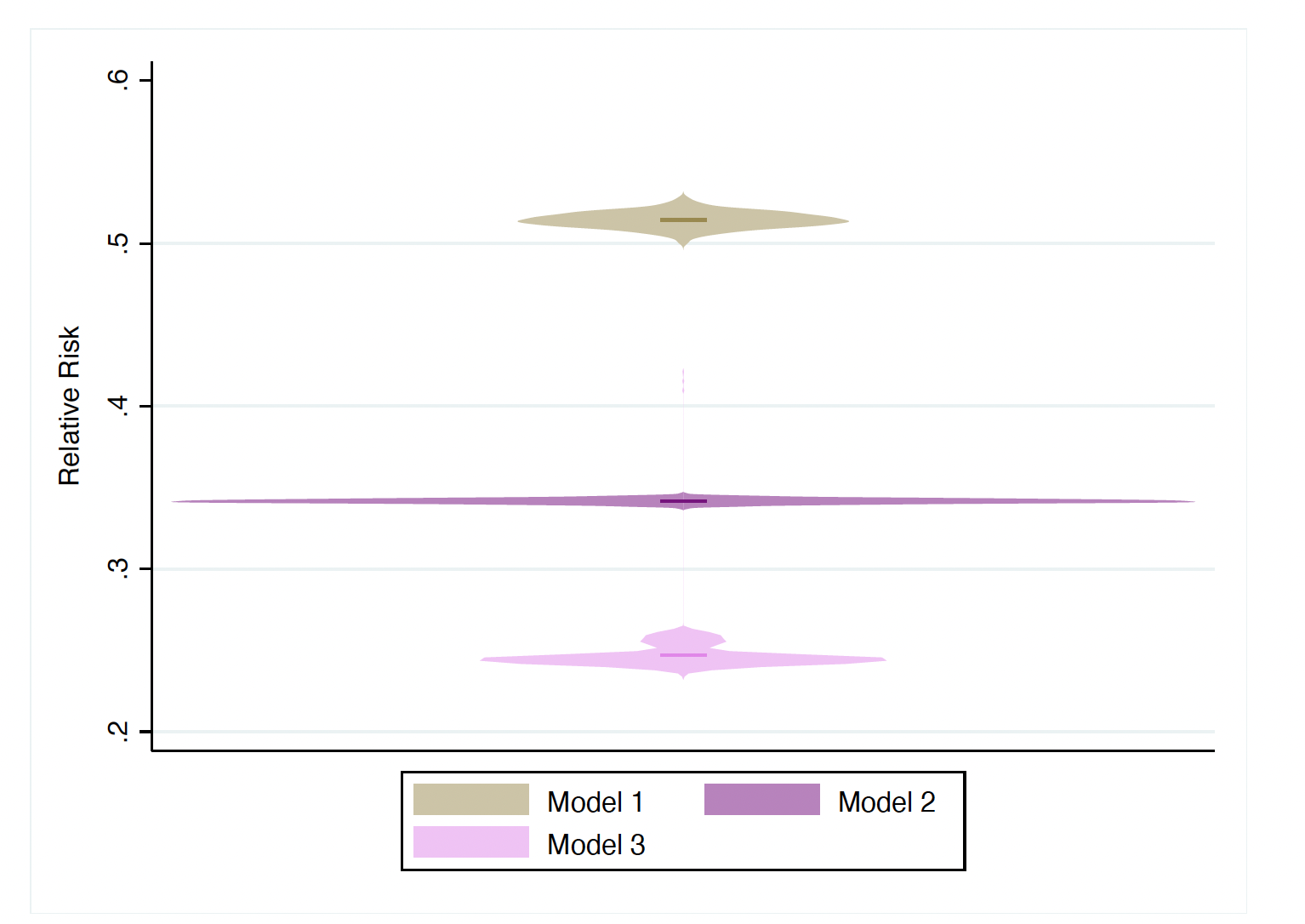
**

**Supplementary Figure 2. Time Varying Mask Mandate Effect**

Estimates of relative risk of SARS-CoV-2 derived from Model 1 with mask mandate effect treated as a time-varying covariate, through multiplicative interaction with a cubic time trend term (purple curve). Risk reduction associated with mask mandates *increases* over time, as would be expected given an exponential reduction in growth associated with reduction of the epidemic reproduction number via mask mandate effects. Gray shaded area represents 95% confidence interval. The pink curve (and associated gray shaded area) represents the relative risk (0.51) and 95% confidence intervals (0.47 to 0.56) for mask mandates derived from Model 1 without time-varying mask effects.
